# Patients’ Preferences and the Time to Finish Gonadotropin-Releasing Hormone (GnRH) Agonist and Antagonist Injections in Japanese Prostate Cancer Patients

**DOI:** 10.7759/cureus.84881

**Published:** 2025-05-27

**Authors:** Takashi Kawahara, Akihito Hasizume, Yasuhide Miyoshi, Daiki Ueno, Masanobu Yamazaki, Jun-ichi Teranishi, Kazuhide Makiyama, Hiroji Uemura

**Affiliations:** 1 Urology, Yokohama City University Medical Center, Yokohama, JPN; 2 Urology and Renal Transplantation, Yokohama City University Medical Center, Yokohama, JPN; 3 Urology, Yokohama City University, Yokohama, JPN

**Keywords:** degarelix, gnrh agonist, gonadotropin-releasing hormone, gonadotropin-releasing hormone (gnrh) antagonist, goserelin acetate, leuprorelin acetate (leuprolide)

## Abstract

Introduction

Currently, androgen deprivation therapy (ADT) plays a key role in treating advanced prostate cancer, particularly in elderly patients. With the advent of gonadotropin-releasing hormone (GnRH) medications, ADT has shifted from surgical to medical castration. While GnRH agonists and antagonists remain mainstream treatments for prostate cancer, there has been no research comparing the burden each drug places on patients.

Methods

A study conducted at Yokohama City University Medical Center analyzed 851 hormonal injections administered between August 2018 and February 2019. The study evaluated the time from prescription to completion of injection, as well as the perceived physical and mental burden on patients. Results showed that injections of degarelix took significantly longer time to injection than other treatments. Leuprorelin 22.5 mg most effectively reduced outpatients' hospital visits, primarily due to its six-month dosing interval and convenient kit formulation. Degarelix required a longer process, including drug dilution and cooling of the injection site to prevent potential skin reactions, contributing to its extended administration time.

Results

In terms of patient burden, leuprorelin 22.5 mg was associated with the least discomfort, showing minimal difference from the oral formulation. One limitation of this study is that the method of leuprorelin administration in Japan (subcutaneous injection) differs from that in other countries (intramuscular injection).

Conclusion

In summary, the six-month leuprorelin 22.5 mg regimen reduces hospital time. Patient burden was also considered a factor in the selection of GnRH preparations.

## Introduction

Huggins et al. first performed surgical castration for prostate cancer in 1941 [1]. Since then, androgen deprivation therapy (ADT) has played an important role in managing prostate cancer. With the recent development of gonadotropin-releasing hormone (GnRH) medications, ADT has shifted from surgical to medical castration. Leuprorelin was first developed in 1985, and goserelin was introduced in 1987. A three-month depot formulation was launched in 1999 [2,3]. These GnRH agonists have been used as ADT for prostate cancer for over four decades. After introducing degarelix, a GnRH antagonist, in Europe and the United States in 2008, it became clinically available in 2018 in Japan, followed by the introduction of a three-month formulation in 2019 [4]. Although both GnRH agonists and antagonists effectively control serum testosterone levels, they differ in dose duration, needle size, and adverse effects, including skin reactions [5]. In the present study, we examined patients’ preferences regarding GnRH agonists and antagonists, as well as differences in the time required to complete their administration in a hospital setting.

This article was previously posted to the medRxiv preprint server on Aug 25, 2023 [6].

## Materials and methods

A total of 851 hormonal injections were administered between August 2018 and February 2019 at Yokohama City University Medical Center (Yokohama, Japan). Inclusion criteria were adult men undergoing hormone therapy for prostate cancer, and exclusion criteria were patients who were seen by an outside clinic on the same day and who had cognitive problems. None of the patients received generic drugs. Nine cases (1.0%) received leuprorelin 3.75 mg (Leuprin®︎), 232 cases (27.3%) received leuprorelin 11.25 mg (Leuprin SR®︎), 187 cases (22.0%) received leuprorelin 22.5 mg (Leuprin PRO®︎), 206 cases (24.2%) received goserelin 10.8 mg (Zoladex LA®︎), and 217 cases (25.5%) received degarelix 80 mg (Gonax®︎). This study was approved by the Institutional Review Board (B200800065), and the need for consent to participate was waived.

All injections were administered by medical nurses subcutaneously after prescription by urologists. The time from ordering GnRH agonists or antagonists to the completion of the injection was analyzed. The total time per year was calculated each time and multiplied by the number of times per year. We also asked 50 patients about the mental and physical loads of hormonal injections. Patients were randomly selected with stable prostate-specific antigen (PSA) and a performance status score of 0 to avoid other factors. The load was evaluated based on the following question: “If the hormonal injection could be changed to an oral tablet form, how much (in Japanese yen (JPY)) would you be willing to pay per dose?’’. The total cost per year was calculated as the one-time cost multiplied by the number per year. These methods were used as previously used in another study [7]. All patients had received the same GnRH treatments more than twice before the study.

The time and cost were analyzed by the Mann-Whitney U test using the GraphPad Prism software version 7.05 (GraphPad Software, San Diego, CA). P-values <0.05 were considered to indicate statistical significance.

## Results

**Figure 1:**
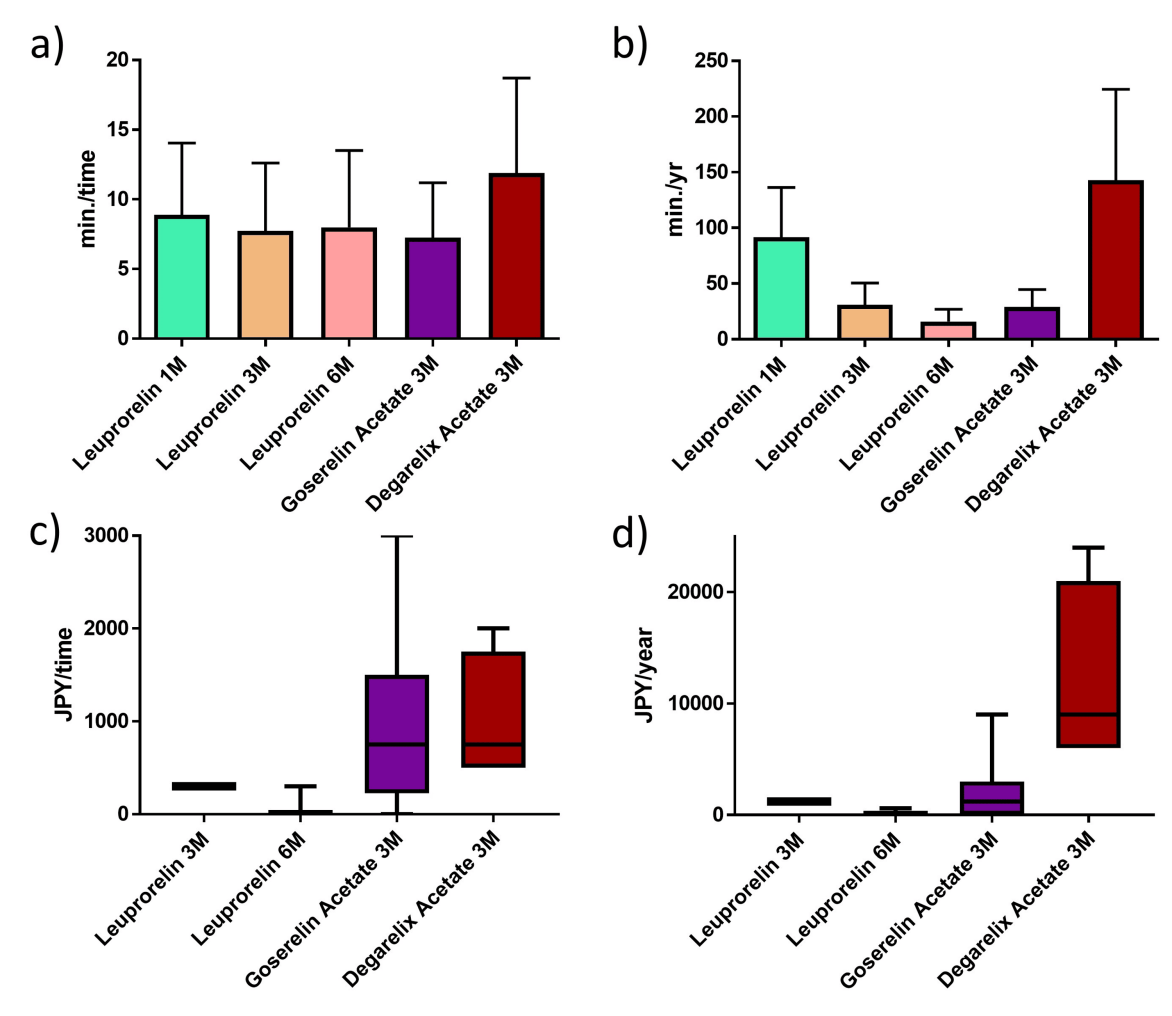
The time for injection and the burden felt by patients. The time from ordering hormonal therapy to the time to finish degarelix injection was significantly longer than that in other GnRH treatments (p < 0.001) (a, b). The median (mean ± SD) cost of load to injection was 300 Japanese yen (JPY) (325.0 ± 35.4) for leuprorelin 11.25 mg, 0.0 (40.0 ± 105.6) JPY for leuprorelin 22.5 mg, 750.0 (966.7 ± 1070.8) JPY for goserelin 10.8 mg, and 750.0 (1000.0 ± 707.1) JPY for degarelix 80 mg (c, d).

The median (mean ± SD) time from ordering hormonal therapy to completion of injection was seven minutes (8.9 ± 1.7) for leuprorelin 3.75 mg, seven minutes (7.7 ± 4.9) for leuprorelin 11.25 mg, eight minutes (8.0 ± 5.5) for leuprorelin 22.5 mg, six minutes (7.3 ± 3.9) for goserelin 10.8 mg, and 10 minutes (11.9 ± 6.8) for degarelix 80 mg. This time included the preparation time for drug dissolution. The time required for degarelix was significantly longer than for the other GnRH treatments (p < 0.001) (Figures 1A, 1B).

The median (mean ± SD) cost of the burden associated with injection was 300 JPY (325.0 ± 35.4) for leuprorelin 11.25 mg, 0 JPY (40.0 ± 105.6) for leuprorelin 22.5 mg, 750 JPY (966.7 ± 1070.8) for goserelin 10.8 mg, and 750 JPY (1000.0 ± 707.1) for degarelix 80 mg.

Among patients receiving leuprorelin 22.5 mg, 13 out of 15 (86.7%) expressed no desire to switch to oral tablets (Figures 1C, 1D).

## Discussion

This study sought to provide a comprehensive evaluation of the patient burden associated with GnRH agents, which remain central to the management of prostate cancer despite the emergence of novel therapeutic agents. Currently available formulations include GnRH agonists such as leuprorelin and goserelin acetate, and the GnRH antagonist degarelix. Although previous studies have addressed issues such as injection site reactions and adverse effects (e.g., skin rashes with degarelix or needle-related pain), few have directly evaluated patient preferences and burden [8-11]. To our knowledge, this study is the first to assess GnRH preparations primarily from the patient’s perspective.

The six-month leuprorelin formulation was associated with the lowest per-injection burden, as well as the lowest cumulative burden over a 12-month period. Notably, 86.7% of patients receiving this formulation indicated a preference to continue with injectable therapy, even if an oral alternative became available. In contrast, only 10.0% of goserelin users and none of the degarelix users expressed such a preference, suggesting a greater perceived burden among these groups. Commonly reported issues included injection-related pain, cutaneous adverse events, and the inconvenience associated with frequent hospital visits. A phase 3 trial in Japan showed injection site pain (76.9%), injection site induration (73.5%), injection site erythema (71.8%), and injection site swelling (28.2%). Miyajima et al. compared the 80 mg and 480 mg formulations of degarelix and found that injection site reactions were 84% for 80 mg and 92% for 480 mg. Redness, induration, swelling, warmth, and itching were the most common side effects of degarelix [10]. Naya et al. reported that in the group using goserelin, 74.1% of patients showed improvement in pain scores when a cooling gel was performed prior to administration [11]. In our case, we do not use cooling for goserelin, but we do use it for degarelix, because cooling before injection improves skin reaction and pain.

Furthermore, the mean duration of in-hospital stay required for administration was evaluated. For three-month leuprorelin, six-month leuprorelin, and three-month goserelin formulations, the average times were 7.44, 7.55, and 7.15 minutes, respectively, demonstrating minimal differences. In contrast, the administration of degarelix required significantly more time due to both the reconstitution procedure and the pre-injection cooling protocol. The cooling protocol is applying an ice pack to the skin of the abdomen for five minutes before the injection. Consequently, the estimated annual in-hospital time was markedly higher for degarelix (142.47 minutes: 11.87 min/time x 12 months) compared to the six-month leuprorelin formulation (15.58 minutes). This outcome is attributable to both its extended six-month dosing interval and its pre-packaged kit formulation. While leuprorelin and goserelin are available as ready-to-use kits, degarelix necessitates manual reconstitution prior to administration, thereby extending the preparation time. In a report by Montgomery et al. comparing goserelin and leuprorelin, goserelin tended to be shorter than leuprorelin in terms of time required for injection [12]. In the present study, we did not find a large difference in time. This may be because our study compared in-hospital length of stay, including injection time, and thus, a small difference in injection time may not have been significant. On the other hand, the in-hospital stay time for degarelix was significantly longer than that for goserelin and leuprorelin because of the time required for skin cooling and dilution. Moreover, due to its potential to induce local skin reactions such as erythema and pain, additional cooling of the injection site is routinely implemented in our clinical setting, further prolonging the administration process.

This discrepancy is clinically relevant in the context of long-term treatment, particularly in adjuvant or neoadjuvant settings, and in patients with metastatic castration-sensitive prostate cancer, who may undergo GnRH therapy for periods exceeding five years.

This study has several limitations. The financial burden was quantified using direct patient costs, but established quality of life instruments, such as EuroQol 5 Dimension (EQ-5D), Functional Assessment of Cancer Therapy - Prostate (FACT-P), and Short Form-36 (SF-36), were not employed. These instruments, while comprehensive, may not adequately capture injection-specific discomfort, such as pain or local adverse effects. Furthermore, the relative impact of individual burden components was not quantified. Nonetheless, the present findings offer a novel perspective on the patient-centered evaluation of GnRH analogues and suggest that consideration of patient burden may inform more individualized therapeutic decision-making in clinical practice. Secondly, in Japan, leuprorelin is administered subcutaneously in accordance with domestic regulatory guidelines, whereas intramuscular administration is standard practice in many other countries. Additionally, since 2019, a three-month regimen of degarelix (240 mg administered in two injections) has become available in Japan. Thus, further investigations incorporating this updated regimen are warranted.

## Conclusions

When considering GnRH preparations in terms of patient burden, the six-month formulation of leuprorelin was the least burdensome treatment for patients. In the selection of GnRH drugs, there are various factors such as cardiovascular events, injection time, prostate volume, and PSA response, but patient burden was considered to be one of the critical factors to be considered. Therefore, it is important for each physician to determine which factors are important in the use of hormone therapy for prostate cancer.
